# Controversies about refrigeration of dental adhesives: a review

**DOI:** 10.1038/s41405-025-00379-9

**Published:** 2025-12-10

**Authors:** Omar Abd El-Maksoud, Hamdi Hosni Hamdan Hamama, Ramy Ahmed Wafaie, Salah Hasab Mahmoud

**Affiliations:** 1 Conservative Dentistry Department, Faculty of Oral and Dental Medicine, Delta University for Science and Technology, Gamasa, Egypt; 2 Conservative Dentistry Department, Faculty of Dentistry, Mansoura University, Mansoura, Egypt; 3 Faculty of Oral and Dental Medicine, Alsalam University, Tanta, Egypt; 4 Faculty of Dentistry, Aqaba Medical Sciences University, Aqaba, Jordan

**Keywords:** Restorative dentistry, Dental materials

## Abstract

**Introduction:**

The refrigeration of dental adhesives is a routine practice, often adopted without thorough understanding or consideration of its scientific basis and potential clinical consequences. This review explores the effects of refrigeration and subsequent immediate use on the bonding effectiveness of dental adhesive systems. Given the ongoing debate in the literature about how storage temperature influences adhesive characteristics such as viscosity, degree of conversion, and bond strength, this work synthesizes findings from both supportive and contradictory studies to inform clearer clinical guidance.

**Methods:**

Relevant literature was identified through a comprehensive search of key databases, including PubMed, Scopus, and Google Scholar, focusing primarily on in-vitro studies that investigated the effect of refrigeration on the bond strength and bonding-related kinetics of various adhesive systems: etch-and-rinse, self-etch, and universal adhesives.

**Results:**

The collected data suggest that immediate use of adhesives post-refrigeration may induce material-dependent adverse changes in viscosity, solvent evaporation, and polymerization kinetics. These changes have primarily been linked to various factors that appear to critically mediate the influence of low temperature, including adhesive composition and solvent type. Although some studies have supported these findings, the anticipated deleterious effects could not be definitively confirmed, as several other investigations have reported negligible or no effects at all across different adhesive systems.

**Conclusions:**

The heterogeneity of findings indicates that no conclusive agreement has been established regarding the actual risks associated with refrigerating dental adhesives. Nonetheless, the absence of evidence supporting any benefit from immediate post-refrigeration use suggests that allowing adhesives to reach room temperature before use remains a cautious and prudent approach.

## Introduction

Adhesive dentistry is a major dental discipline that relies on establishing a reliable bond and a durable seal between the restoration and the tooth structure using adhesive materials [1–3]. Dental adhesive systems have gained global popularity and attracted significant research attention, paving the way for minimally invasive techniques and conservative restorative approaches [4, 5]. Traditionally, dental adhesives were classified into seven sequential generations based on their development timeline. The fourth generation, known as the "three-step etch-and-rinse" adhesive, was considered the "gold standard" [6]. Subsequent generations were introduced up to the seventh generation, known as "all-in-one self-etch adhesives," aimed at enhancing clinical efficiency by reducing the application steps and complexity in technique [7, 8].

Subsequently, a new classification was introduced by Van Meerbeek in the early 2000s [9]. According to this classification, current adhesive systems were classified into two different categories based on their interaction with tooth substrates: etch-and-rinse systems, which involve removing the smear layer, and self-etch systems, which incorporate the smear layer into the hybrid substrate [7, 10]. A primary distinction lies in the use of a separate phosphoric acid etching step (typically 35-37% phosphoric acid) in etch-and-rinse systems [11]. Based on the number of clinical application steps, etch-and-rinse adhesives are further classified into: (a) three-step and (b) two-step etch-and-rinse adhesives. Similarly, self-etch adhesives are classified as (a) two-step and (b) one-step self-etch adhesives [6, 12]. Classified within the latter category, universal adhesives were later introduced as multi-mode systems, offering fewer application steps and versatile adhesion strategies, owing to their capability for use with etch-and-rinse, self-etch, and selective enamel etching techniques [10, 13, 14]. Irrespective of their category, all dental adhesives share a similar general composition. They typically contain hydrophobic and hydrophilic monomers, volatile solvents, photoinitiators, and co-initiator systems, with each component playing an essential role in determining the final performance of the adhesive [15].

The ongoing advancements in adhesive systems have fostered a wide diversity of adhesion modalities [13, 16]. Nevertheless, achieving an optimal and long-term bond between the tooth structure and restoration remains a persistent challenge [11, 17]. The variations in bonding mechanisms and chemical compositions significantly influence the effectiveness of dental adhesives [18]. However, several other factors, mainly related to clinical handling, have also been identified to affect their final performance, such as conditioning time [2], method of solvent removal [19], environmental humidity [20], and curing efficiency [21]. One factor that is frequently overlooked, yet critically important is the pre-cure temperature [22]. The pre-cure temperature of adhesive systems is considered a key factor influencing the rheological, chemical, physical and mechanical behavior of dental adhesives [2]. It can influence properties such as viscosity, degree of conversion, solvent evaporation, penetration ability, all of which are crucial for optimal bond strength and durability [23, 24]. Therefore, attention to the pre-cure temperature of dental adhesives, typically represented by their storage temperature, holds utmost clinical significance. However, many dental practitioners often do not take into consideration the potential effect this factor may have on the final performance of dental adhesives.

The launch of dental adhesives to the market requires confirming that they can be stored for a long time without deterioration in stability or functionality to avoid any adverse effects on their bonding performance and clinical safety [1]. However, these instructions are generalized and often do not reflect the material’s sensitivity to temperature changes over time. While some manufacturers recommend room temperature storage, others recommend refrigeration at 2–5 °C [16, 25], yet provide no clear guidance on the ideal time for use after refrigeration or on optimal post-storage handling. Etch-and-rinse adhesives were thought not to require refrigeration [26]. Meanwhile, concerns about the stability of the complex formulations of self-etch and universal adhesives have supported recommendations for refrigeration to slow chemical deterioration, reduce hydrolytic degradation, and extend their shelf life [1, 16, 27, 28].

Storing adhesives in refrigerators has become a widespread practice, particularly in warm areas or hot climates with a belief that such approach helps prevent premature solvent evaporation and subsequent deterioration of adhesive formulation [1, 29]. These consequences are believed to be more pronounced when adhesives are stored above room temperature, such as near ovens or other heat-emitting devices. Elevated temperatures may influence chemical reactions, thereby compromising the quality and durability of the bond [22]. This, in turn, has reinforced the refrigeration of dental adhesives, regardless of their category, as a precautionary measure.

Occasionally, many clinicians who refrigerate adhesives use them immediately after removal without allowing time for them to reach room temperature [25]. This practice, however, brings to mind an important question: Can this approach be considered appropriate, given that low temperatures increase viscosity, potentially restricting adhesive infiltration and compromising clinical efficacy? The raised question about this routine practice makes it a matter of significant interest, with growing uncertainty about its suitability for dental adhesives and whether they should be allowed to reach room temperature prior to application. Therefore, the aim of this work was to assess how refrigerator storage and immediate use thereafter affect the bonding effectiveness of dental adhesive systems. In this sense, relevant studies addressing this aspect were highlighted.

## Methods

A comprehensive search was performed to explore the existing literature on the effects of refrigeration and subsequent immediate use on the bonding effectiveness of dental adhesive systems. The search was conducted using PubMed, Scopus, and Google Scholar databases between 23 June and 2 July 2025 to identify relevant studies available in English language, with no restrictions on the year of publication.

The inclusion criteria were as follows: (1) original in-vitro peer-reviewed studies; (2) studies testing the effect of low temperature or refrigeration on the bond strength, viscosity, degree of conversion, or penetration ability of dental adhesives; and (3) studies clearly reporting methodological details regarding adhesive brand/type, temperature conditions, and testing method. Exclusion criteria included non-English papers without full English text, clinical studies, all forms of reviews including systematic reviews and meta-analyses, conference proceedings, case reports, case series, letters to the editor, editorials, and policy documents. Moreover, in-vitro studies unrelated to refrigeration or low-temperature effects and studies involving experimental adhesives were also excluded. The following keywords and their combinations were used in the search process: “refrigeration” OR “refrigerator storage” OR “low temperature” OR “room temperature” AND “shear bond strength” OR “tensile bond strength” OR “bonding durability” OR “bonding performance” AND “adhesive systems” OR “bonding agents” AND “etch-and-rinse” OR “self-etch” OR “universal”. The search also included cross-referencing citations from key articles to identify additional relevant studies.

**Fig. 1:**
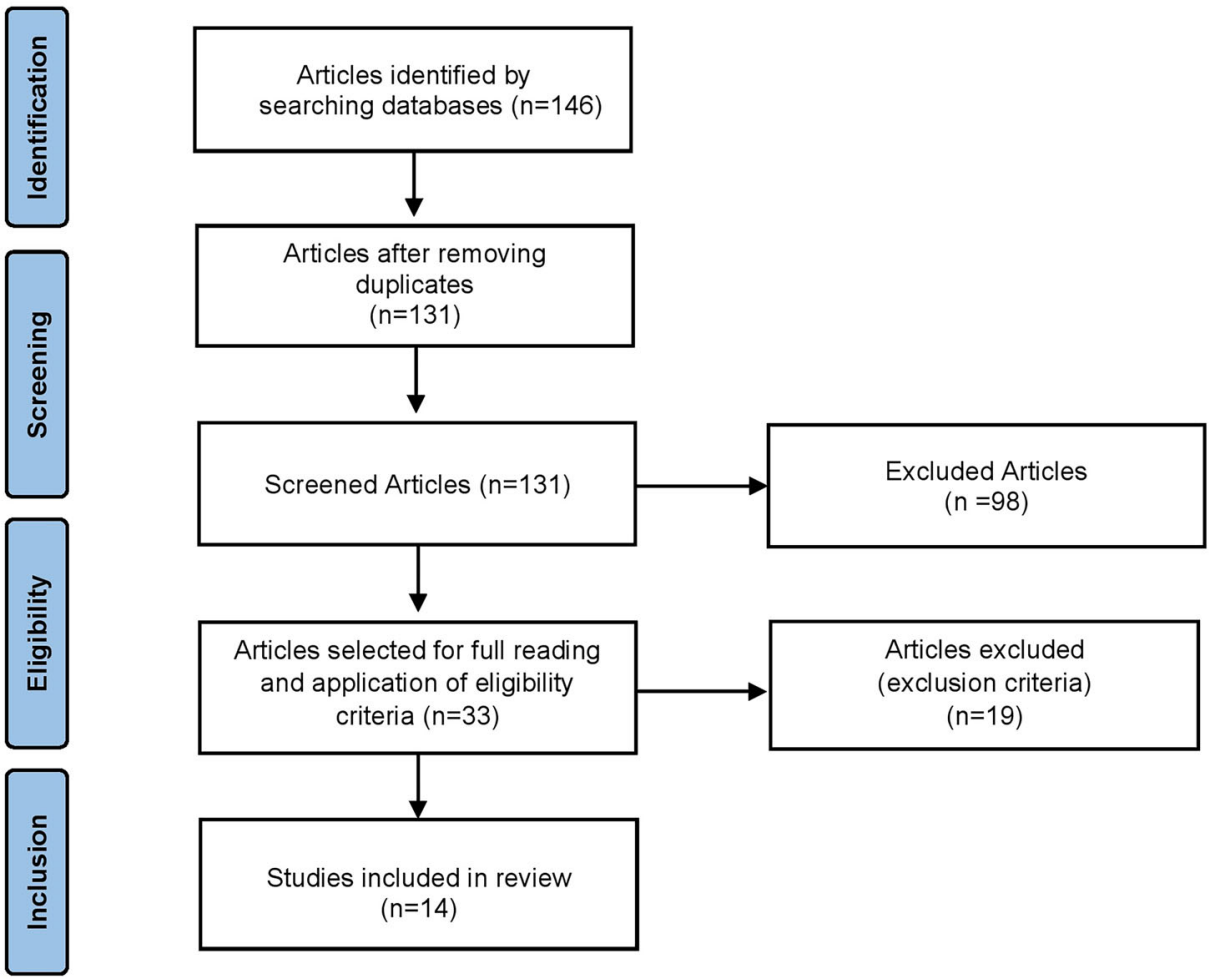
Flowchart illustrating the study selection process.

A total of 146 records were initially retrieved, of which 15 duplicates were removed. The remaining 131 articles underwent title and abstract screening, and 98 were excluded due to their irrelevance. Thereafter, 33 full-text articles were reviewed for eligibility, and 19 were excluded for not fulfilling the inclusion criteria. Finally, 14 studies published between 1999 and 2022 met the eligibility criteria and were included. The selection process is illustrated in Fig. 1. The included studies examined the effects of refrigeration or low-temperature storage on the bond strength and bonding-related properties of different adhesive systems. A summary of studies included in the review is presented in Table 1.

**Table 1:** Summary of studies included in the review

Author	Year	Materials	Objective	Outcome
1- Hagge et al. [43]	1999	• Prime & Bond (Dentsply/Caulk, Milford, Delaware, USA) • All-Bond 2 (Bisco Inc, Shaumburg, Ill, USA) • Scotchbond Multi-Purpose (3M Dental Products Division, St. Paul, MN, USA)	• To investigate the shear bond strength of different adhesive systems applied at refrigerated and room temperatures.	• Refrigeration had no significant effect on the shear bond strength of Prime & Bond or All-Bond 2. • Shear bond strength of Scotchbond Multi-Purpose was higher at refrigerated temperature than at room temperature.
2- Spohr et al. [33]	2001	• Scotchbond Multi-Purpose (3M Dental Products Division, St. Paul, MN, USA) • Single Bond 3M Dental Products Division, St. Paul, MN, USA) • Prime & Bond NT (Dentsply/Caulk, York, PA, USA)	• To investigate the tensile bond strengths of three etch-and-rinse adhesive systems applied to dentin at refrigerated and room temperatures.	• The tensile bond strengths were statistically similar when all adhesive systems were applied at refrigerated and room temperatures • No adverse effects upon tensile bond strength were observed when adhesive systems were taken directly from refrigerated storage.
3- Borges et al. [25]	2006	• AdheSE (Ivoclar Vivadent, Shaan, Liechtenstein) • Clearfil SE Bond (Kuraray Corp.,Tokyo, Japan) • One-Up Bond F (Tokuyama Corp.,Tokyo, Japan)	• To investigate the tensile bond strength to dentin of three self-etching adhesive systems at refrigerated and room temperatures.	• No statistically significant differences in tensile bond strength were found between the adhesive systems applied at refrigerated and room temperatures. • No adverse effects on tensile bond strength were observed when self-etching adhesive systems were used after being taken directly from the refrigerated storage.
4- Sundfeld et al. [39]	2006	• Adper Prompt L-Pop (3M ESPE Dental Products, St Paul, MN, USA)	• To investigate the penetration of an aggressive self-etching adhesive system at refrigerated and room temperatures into ground and unground enamel surfaces.	• The self-etch adhesive exhibited significantly lower enamel penetration at 6 °C compared to room temperature (25 °C) and to 30 min after removal from the refrigerator.
5- Sadr et al. [1]	2007	• Clearfil Tri-S (Kuraray Medical, Osaka, Japan) • Clearfil SE Bond (Kuraray Medical, Osaka, Japan)	• To evaluate the micro-shear bond strength to enamel and dentine, pH and hardness of two-self etching adhesives over a period of 60 weeks at 4, 23 or 37 °C.	• No significant difference in the microshear bond strength was observed for either adhesive when stored at room or refrigerated temperatures. • To assure an optimum performance, it is advisable that the clinicians store these materials in the refrigerator, as the manufacturer instructs, particularly in the warmer areas or seasons.
6- de Alexandre et al. [22]	2008	• Prime&Bond NT (Dentsply Caulk, Milford, DE, USA) • Adper Prompt L-Pop (3M ESPE Dental Products, St Paul, MN, USA) • Clearfil SE Bond (Kuraray Medical Inc, Kurashiki, Japan).	• To evaluate the microtensile bond strength, micromorphology of resin-enamel interface and etching patterns promoted by an etch-and-rinse adhesive, and two self-etching adhesives, to ground bovine enamel surfaces, when applied at temperatures of 5 °C, 40 °C, and 20 °C.	• The cold temperature of the tested adhesives did not present a significant reduction in the microtesnile bond compared to room and high temperatures.
7- Donmez et al. [16]	2009	• Clearfil SE Bond (Kuraray Medical Inc., Tokyo, Japan)	• To evaluate the effect of storage temperature on microtensile bond strength of a self-etching primer system to pulp chamber dentin.	• Keeping the adhesive system in refrigerator or room temperature did not affect microtensile bond strength compared to the control group (immediately-delivered from the manufacturer). • SE Bond adhesive system should be kept in refrigerator until expiry date.
8- Reis et al. [23]	2009	• Adper Single Bond 2 (3M ESPE, St Paul, MN, USA) • Prime& Bond 2.1 (Dentsply De Trey, Konstanz, Germany)	• To test the effect of adhesive temperature on the microtensile bond strength to dentin and silver nitrate uptake of an ethanol/water and an acetone-based etch-and-rinse adhesive system.	• No significant difference in microtensile bond strength was detected for both adhesives at 5 °C and 20 °C.
9- Faria-e-Silva et al. [2]	2010	• Scotchbond Dual-Cure (3M ESPE, St. Paul, MN, USA) • Clearfil SE Bond (Kuraray, Tokyo, Japan)	• To evaluate the effect of refrigeration at 4 °C and post-refrigeration times (immediate, 5, 10, 15, or 20 min) on the viscosity and conversion kinetics of dental adhesive resins.	• Refrigeration presented a significant time- and material-dependent effect on the viscosity and polymerization kinetics of the dental adhesives. • Adhesive agents should be removed from the refrigerator at least 20 min before being used.
10- Graham & Vandewalle. [26]	2010	• iBond (Heraeus Kulzer, SouthBend, IN) • Clearfil SE Bond (Kuraray, NewYork, NY).	• To compare the shear-bond strength of composite resin to dentin using two different self-etching adhesives after extended storage at room or refrigerated temperatures.	• Refrigerated or room-temperature storage of the one- or two-step adhesive bonding agent did not affect the shear bond strength of composite resin to dentin.
11- Loguercio et al. [24]	2011	• Prime&Bond 2.1 (Dentsply De Trey, Konztanz, Germany) • Adper Single Bond (3M ESPE, St. Paul, MN, USA)	• To evaluate the effect of adhesive temperature on the resin-dentin bond strength, nanoleakage, adhesive layer thickness, and degree of conversion of ethanol/water- and acetone-based etch-and-rinse adhesive systems.	• No significant difference in terms of microtensile bond strength, degree of conversion, and adhesive layer thickness between the refrigerated temperature (5 °C) and the room temperature (20 °C) • It does not matter if the refrigerated product is used soon after its removal from the refrigerator or after it reaches room temperature.
12-Sharafeddin et al. [30]	2015	• Adper Single Bond (3M ESPE, St. Paul, MN, USA) • Clearfil SE Bond (Kuraray Medical Inc., Kurashiki, Japan)	• This study aimed to evaluate the effect of temperature on shear bond strength of etch-and-rinse and self-etching adhesives to ground bovine dentin surfaces, at temperatures of 4 °C, 25 °C and 40 °C.	• Low temperature significantly decreased the shear bond strength of the etch-and-rinse adhesive compared to higher temperatures; therefore, storing this adhesive out of refrigerator could yield better clinical results. • Refrigeration of the tested self-etch adhesive did not induce a significant difference in the bond strength when compared to other temperatures indicating that the storage temperature before using this adhesive creates no difference.
13- Akarsu & Aktuğ [45]	2020	• Universal Single Bond (3M ESPE, St. Paul, MN,USA) • All Bond Universal (Bisco Inc, Shaumburg, Ill, USA) • Clearfil SE Bond (Kuraray Noritake Dental Inc., Osaka, Japan)	• To evaluate the shear bond strength of two universal adhesives and a two-step self-etch adhesive system to dentine at various temperatures (4 °C, 20 °C, 36 °C, 55ºC).	• No statistically significant differences were observed in comparing the shear bond strength of the refrigerated and room-temperature conditions across all tested adhesives.
14- Yumitate et al. [12]	2022	• Tokuyama Universal Bond (Tokuyama Dental, Tokyo, Japan)	• To evaluate the influence of tooth and adhesive temperature during the bonding procedure on the effectiveness of dentin bonding.	• The temperature of the material does not affect bonding effectiveness in terms of microtensile bond strength. • It does not matter if the refrigerated product is used soon after its taking from the refrigerator or after it reaches room temperature in the clinic.

### Theoretical drawbacks of refrigeration

Questioning the immediate use of refrigerated adhesives, the potential drawbacks may theoretically center on the influence of low temperature on the viscosity of the adhesives. One of the most distinctive characteristics of adhesive systems is their low viscosity, which is achieved through the addition of solvents and diluent monomers, allowing excellent volatilization and deep substrate penetration [22, 30]. Low temperature may dramatically increase the material viscosity. It was reported that the higher the viscosity of an adhesive, the more challenging is the substrate wetting [31], owning to the reduced spreading velocity [32]. Therefore, high viscosity could hinder proper fluidity and impair the full- infiltration of the adhesive into the etched tooth substrate [24].

Moreover, given that adhesion depends not only on the successful permeation of the adhesive into the dental substrate, but also on the mechanical properties and quality of the final polymer [10], the kinetic energy of the monomer molecules is thought to be decreased at low temperatures, and thereby reducing collision frequency during the polymerization reaction [2, 24, 33]. This may result in incomplete polymerization and subsequently affect certain properties related to polymerization efficiency, such as microhardness and diametral tensile strength [24, 25, 33].

Another plausible consequence of post-refrigeration immediate use is primarily related to solvent evaporation [25]. The monomers in dental adhesives are typically solvated in volatile organic solvents and water. Solvent constituents of adhesive systems, such as ethanol, acetone and water, can reach up to 80% of the formulation by weight [15, 34–36], and should ideally be removed to a sufficient degree during adhesive application [10]. Refrigeration may affect solvent vapor pressure, hindering the complete evaporation of the solvents from the adhesive layer [37]. The presence of a high amount of residual solvent entrapped inside the adhesive layer may further inhibit the polymerization, negatively affect the mechanical properties of the adhesive, reduce the bond strength and deteriorate the sealing ability of the adhesive system [10, 22, 38].

### Evidence from literature: supportive versus contradictory studies

Supporting these concerns, several studies have reported an underperformance of refrigerated adhesives in terms of viscosity, degree of conversion and penetration ability. Faria-e-Silva et al. [2] evaluated the effect of refrigeration and different post-refrigeration times on the viscosity and conversion kinetics of two different adhesive systems (Scotchbond Dual-Cure; 3M ESPE and Clearfil SE Bond; Kuraray). Their findings revealed that the refrigeration of adhesives induced a significant, time- and material-dependent effect. A noticeable increase in viscosity was observed in both adhesives in response to the low-temperature effect, which could potentially reduce molecular mobility and consequently hinder the material’s ability to flow. Notably, the viscosity values did not return to levels similar to those of their non-refrigerated counterparts until 20 min after removal from the refrigerator.

Both tested adhesives were adversely affected, yet they exhibited different responses in terms of the speed of temperature rebound, as well as the extent of viscosity increase and its subsequent recovery over time. These observations were attributed to compositional differences between the formulations. Both adhesives contain the highly viscous bisphenol-A glycidyl dimethacrylate (Bis-GMA) monomer. However, the diluent monomer in Scotchbond is triethylene glycol dimethacrylate (TEGDMA), whereas Clearfil contains hydroxyethyl methacrylate (HEMA), which has lower viscosity. Differences in the Bis-GMA-to-diluent ratio represent another factor that may account for the variation in viscosity. The higher Bis-GMA content in Scotchbond could explain the increased viscosity observed during refrigeration compared with Clearfil. Additionally, the presence of fillers increased the viscosity of Clearfil, causing its fluidity to recover more slowly than that of Scotchbond. These findings therefore, reflect a clearly material-dependent response. Moreover, refrigeration also significantly reduced the degree of conversion assessed immediately and at 5 min after refrigeration for both adhesives. This reduction was also attributed to the material-specific variation in rheological behavior, which likely restricted monomer mobility and, in turn, slowed the polymerization rate and the degree of conversion. As a final conclusion, the study encouraged waiting at least 20 min after refrigerating adhesives before clinical use.

Another study reflecting the undesired consequences of refrigeration was conducted by Sundfeld et al. [39]. In this study, the ability of a self-etch adhesive (Adper Prompt L-Pop; 3M ESPE) to penetrate ground and unground enamel surfaces was assessed at room temperature, immediately after removal from the refrigerator (6 °C), and 30 min after removal. Based on the evaluation of penetration depth, the results revealed that the self-etch adhesive exhibited significantly lower enamel penetration at 6 °C compared to both room temperature (25 °C) and the 30-minute post-removal condition. These findings suggest that the low-temperature of refrigerated adhesives may impair their fluidity by increasing viscosity, which in turn could restrict their penetration ability and compromise their overall performance. The aforementioned studies, which confirm the adverse effects of using adhesives immediately after refrigeration, were consistent with several studies [40–42], which reported similar effects in refrigerated composite materials regarding different evaluated aspects such as degree of conversion, microhardness, and marginal adaptation.

However, by reviewing the literature, there seems to be a lack of consensus among researchers regarding this underperformance associated with refrigeration. For instance, Sharafeddin et al. [30] reported contradictory results when assessing the effect of temperature on the shear bond strength of an etch-and-rinse adhesive (Adper Single Bond; 3M ESPE) and a self-etch adhesive (Clearfil SE Bond; Kuraray). The findings showed that low temperature decreased the shear bond strength of the etch-and-rinse adhesive compared to higher temperatures. This could be attributed to the possibility that low temperature may have increased adhesive viscosity, interfering with substrate wetting and limiting co-monomer penetration into demineralized dentin. Additionally, the adhesive layer at low temperature may have become thicker and more variable, contributing to decreased bond strength. On the contrary, refrigeration of the self-etch adhesive did not result in a significant difference in shear bond strength when compared to room-temperature storage. Moreover, the bond strength of the refrigerated self-etch adhesive was significantly higher than that of the refrigerated etch-and-rinse adhesive. These findings were ascribed to compositional differences between the two systems, particularly in solvent type and monomer composition, as well as dynamic variations in solvent evaporation and viscosity. More specifically, the tested self-etch adhesive, Clearfil SE, contains water as its primary solvent, whereas Adper Single Bond contains both ethanol and water. This difference likely influenced evaporation kinetics, consequently affecting the level of water and residual solvent in the adhesive layer and thereby the bond strength. Moreover, because SE primer contains low-viscosity monomers and high hydrophilic concentration, the impact of low temperature may have been minimized, preventing a notable reduction in bond strength. The results emphasized that each adhesive system exhibited a distinct response, further supporting the idea that the effects are material-specific rather than generalizable.

Adding further contradictions, some studies even reported no discernible deleterious effects from refrigerator storage or the immediate use thereafter, irrespective of the adhesive system or compositional variations. Spohr et al. [33] upon evaluating the tensile bond strength of three different etch-and-rinse adhesive systems (Scotchbond Multi-Purpose, Single Bond; 3M, and Prime & Bond NT; Dentsply), found no significant differences between the refrigerated adhesives and their counterparts stored at room temperature. The type of solvents, as well as the extent to which these solvents are evaporated, are considered to be important determinants of bond strength. However, the results of this study indicated that incomplete solvent evaporation likely did not occur with refrigeration, despite the tested adhesives containing different solvents, where Scotchbond Multi-Purpose contains only water in its primer, Single Bond contains water and ethanol, and Prime & Bond NT contains acetone. Additionally, the anticipated decrease in bond strength due to potential adverse effects of refrigeration on the adhesives’ physical properties did not appear to occur. Furthermore, refrigeration did not notably affect clinical handling, despite insignificant increases in viscosity and slight dispensing challenges. Therefore, the study concluded that no adverse effects occur when adhesive systems are used directly after refrigerated storage.

Hagge et al. [43] also evaluated the shear bond strength of three different etch-and-rinse adhesive systems applied at refrigerated and room temperatures and likewise reported no adverse effects from refrigeration. The findings revealed no significant differences in shear bond strength for two of the tested adhesives, Prime & Bond and All-Bond 2, at either temperature. Interestingly, the shear bond strength of the third adhesive, Scotchbond Multi-Purpose, was significantly higher at the refrigerated temperature compared to room temperature. However, the authors did not provide a satisfactory explanation for the enhanced bonding performance of Scotchbond Multi-Purpose stored at 4 °C.

In parallel, Reis et al. [23] reported no significant difference in the microtensile bond strength when examining the effect of temperature on an etch-and-rinse, ethanol/water-based adhesive (Adper Single Bond 2) and an acetone-based adhesive (Prime & Bond 2.1) at 5 °C and 20 °C. This finding was not interpreted as discounting the potential influence of low temperature, but rather as an effect possibly masked by the rapid warming of the adhesives after removal from the refrigerator. The authors observed that during application onto the tooth surface, the adhesives were no longer at 5 °C but had already warmed to 14 °C and 18.3 °C for Adper Single Bond 2 and Prime & Bond 2.1, respectively. Furthermore, the authors attributed the comparable bond strength between the 5 °C and 20 °C groups to the temperature rise caused by the unavoidable heat from the light-curing units [44], to which the adhesives were sufficiently exposed. In the same context, Loguercio et al. [24]. observed no significant differences in microtensile bond strength, degree of conversion, or adhesive layer thickness for the same two different solvent-based etch-and-rinse adhesives tested at 5 °C and 20 °C, also failing to experimentally confirm the theoretical drawbacks of refrigeration, which suggests that it may not matter whether refrigerated adhesives are used immediately or after reaching room temperature.

The findings from those previous studies might imply that the apparent insensitivity to cold temperatures could be specific only to etch-and-rinse systems. Nevertheless, Borges et al. [25] also reported similar outcomes regarding the immediate use of refrigerated self-etch adhesives when investigating the tensile bond strength of two two-step self-etch adhesives (AdheSE; Ivoclar Vivadent and Clearfil SE Bond) and a one-step self-etch adhesive (One-Up Bond F; Tokuyama Corp.) at both refrigerated and room temperatures. The results of the study further indicated that, despite differences in solvent constituents, where both two-step adhesives were water-based while the all-in-one adhesive was acetone-based, refrigeration had no apparent significant influence on solvent evaporation in any of the tested adhesives. Moreover, the absence of adverse effects was presumably attributed to the application time likely being sufficient to avert the anticipated low-temperature effect on the adhesives. This could have minimized any potential decline in their physical properties and the subsequent reduction in bond strength. Similarly, Sadr et al. [1]. when examining the effects of storage temperature on the properties of a one-step (Clearfil Tri-S) and a two-step self-etch adhesive (Clearfil SE Bond), the authors found no significant difference in the microshear bond strength for either adhesive when stored at room or refrigerated temperatures. The study concluded that impactful deterioration was likely to occur over time only in water-containing adhesives and at temperatures exceeding room temperature, further reinforcing the practice of refrigeration following the manufacturer's instructions, especially in warmer regions or seasons.

In accordance with the previous findings, Graham and Vandewalle [26] reported similar findings upon evaluating the effect of long-term storage temperatures on the shear bond strength of two different self-etch adhesives. Their findings indicated that refrigeration and room temperature storage had no significant impact on the shear bond strength of the two-step adhesive (Clearfil SE Bond) or the one-step adhesive (iBond; Heraeus Kulzer). The latter adhesive, iBond, demonstrated a significant decline in bond strength over extended storage times. This deterioration was product specific, as its components, including water, 4-methacryloyloxyethyl trimellitic acid (4-META) and cross-linking monomers are mixed together in a single-bottle formulation, which may increase its susceptibility to hydrolytic degradation. Nevertheless, the reduction in the shear bond strength was time-related rather than temperature-related, as no significant differences were observed between room and refrigerated conditions across all storage times.

In agreement with these studies, Donmez et al. [16] also investigated the effect of storage temperature on the microtensile bond strength of the self etching primer of a two-step adhesive system (Clearfil SE Bond) and reported no significant difference between room temperature and refrigerated storage conditions. The study demonstrated that the storage temperature could have influenced the components of the self-etch primer system, such as methacryloyloxydecyl dihydrogen phosphate (MDP), water, and HEMA. In addition, the hydrolysis of HEMA into methacrylic acid and ethylene glycol under acidic conditions has been reported to be influenced by the storage temperature. However, this effect was observed only at elevated temperatures, as incubation at 40 °C resulted in a decline in bond strength. In contrast, storage in the refrigerator at 4 °C did not induce any significant reduction in bond strength compared to room-temperature storage. This finding led the authors to consider refrigeration as the most appropriate storage method for the SE Bond adhesive.

In further agreement, Yumitate et al. [12]. reached a similar conclusion when investigating the effect of temperature during the bonding procedures on a novel two-bottle self-curing universal adhesive (Tokuyama Universal Bond; Tokuyama Dental). The study evaluated the influence of both tooth and adhesive temperatures on the dentin bonding durability by assessing microtensile bond strength and analyzing failure modes. The results indicated that the dentin bonding ability was indeed influenced by the temperature of the tooth. This was attributed to the effect of low tooth temperature, under which polymerization may be insufficiently promoted and fail to proceed effectively. Consequently, many bubbles within the resin side of the adhesive were observed, likely resulting from residual solvent retained as an adverse effect of insufficient polymerization or water uptake from the dentin side due to delayed polymerization. Since the adhesive was affected by the tooth temperature, the low temperature of the adhesive itself was expected to affect the adhesive properties. However, no significant adverse effects were observed with refrigeration, even when the adhesive was used immediately after removal from the refrigerator. The study concluded that bonding effectiveness was affected by the temperature of the tooth but not by the temperature of the adhesive itself. In other words, it does not matter whether the refrigerated adhesive is used soon after removal from the refrigerator or after reaching room temperature.

Supporting cumulative evidence on different adhesive systems, de Alexandre et al. [22] by assessing the influence of temperature on three different adhesive systems, also reported comparable findings. Upon evaluating the effect of temperature on the microtensile bond strength of an etch-and-rinse adhesive (Prime & Bond NT) and two self-etch systems (Adper Prompt L-Pop and Clearfil SE Bond), the results indicated a statistical difference when comparing the cold conditions of the three tested adhesives. The highest microtensile bond strength was observed with Clearfil SE Bond, owing to its two-bottle presentation and application mode. Individual and active application of the SE primer, followed by the waiting time before application of the adhesive, might have facilitated solvent evaporation, even at low temperatures. Conversely, the tested etch-and-rinse adhesive, Prime & Bond NT, showed lower bond strength and exhibited porosities at the enamel–resin interface. This suggested the presence of residual water and solvents, likely due to reduced solvent and water evaporation that low temperature may have induced. Nonetheless, and irrespective of these findings, no significant differences in microtensile bond strength were found between cold and room-temperature conditions for all tested adhesives.

Consistently, Akarsu and Aktuğ [45] evaluated the effect of temperature on the shear bond strength of two universal adhesives (Universal Single Bond; 3M ESPE and All-Bond Universal; Bisco Inc) and a two-step self-etch adhesive (Clearfil SE Bond). All tested adhesives exhibited low shear bond strength values at 4 °C. This was justified by the increased viscosity and reduced spreading velocity likely occurs at low temperatures, along with decreased vapor pressure of the incorporated solvents, which limits their evaporation from the adhesive layer. Despite these findings, the significantly lower bond strengths of refrigerated adhesives were only evident when compared to their counterparts applied at higher temperatures (36 °C and 55 °C), while no significant differences were observed between the refrigerated and room-temperature conditions across all tested adhesives.

### Ethical approval

Ethical approval and consent were not applicable since this manuscript is a review of the existing literature.

## Conclusions

The effect of refrigeration on dental adhesives remains controversial, with the main concern lying not in refrigeration itself but rather in the immediate use thereafter. Current evidence reveals outcomes varying from material-dependent adverse effects to no observable impact on bonding effectiveness. Most studies, however, failed to establish any clear advantage when adhesives are used immediately after refrigeration. Therefore, allowing adhesives to reach room temperature before application appears to be a more prudent and safer approach to avoid any potential complications.

## Data Availability

The datasets used and/or analysed during the current study are available from the corresponding author on reasonable request.

## References

[1] Sadr A, Ghasemi A, Shimada Y, Tagami J. Effects of storage time and temperature on the properties of two self-etching systems. J Dent. 2007;35:218–25. 16996191 10.1016/j.jdent.2006.08.004

[2] Faria-e-Silva AL, Piva E, Moraes RR. Time-dependent effect of refrigeration on viscosity and conversion kinetics of dental adhesive resins. Eur J Dent. 2010;4:150–5. 20396445 PMC2853827

[3] Milia E, Cumbo E, Cardoso RJ, Gallina G. Current dental adhesives systems. A narrative review. Curr Pharm Des. 2012;18:5542–52. 22632386 10.2174/138161212803307491

[4] Sofan E, Sofan A, Palaia G, Tenore G, Romeo U, Migliau G. Classification review of dental adhesive systems: from the IV generation to the universal type. Ann Stomatol. 2017;8:1–17. 10.11138/ads/2017.8.1.001 PMC5507161 28736601

[5] Mante FK, Ozer F, Walter R, Atlas AM, Saleh N, Dietschi D, et al. The current state of adhesive dentistry: a guide for clinical practice. Compend Contin Educ Dent. 2013;34:2–8. 24571402

[6] Elkaffas AA, Hamama HHH, Mahmoud SH. Do universal adhesives promote bonding to dentin? A systematic review and meta-analysis. Restor Dent Endod. 2018;43:e29. 30135848 10.5395/rde.2018.43.e29 PMC6103541

[7] Vale MR, Afonso FA, Borges BC, Freitas AC Jr, Farias-Neto A, Almeida EO, et al. Preheating impact on the degree of conversion and water sorption/solubility of selected single-bottle adhesive systems. Oper Dent. 2014;39:637–43. 24819598 10.2341/13-201-L

[8] Hiraishi N, Breschi L, Prati C, Ferrari M, Tagami J, King NM. Technique sensitivity associated with air-drying of HEMA-free, single-bottle, one-step self-etch adhesives. Dent Mater. 2007;23:498–505. 16690113 10.1016/j.dental.2006.03.007

[9] Van Meerbeek B, De Munck J, Yoshida Y, Inoue S, Vargas M, Vijay P, et al. Buonocore memorial lecture. Adhes Enamel Dentin: Curr Status Future Chall Oper Dent. 2003;28:215–35. 12760693

[10] Sutil BGDS, Susin AH. Dentin pretreatment and adhesive temperature as affecting factors on bond strength of a universal adhesive system. J Appl Oral Sci. 2017;25:533–40. 29069151 10.1590/1678-7757-2016-0500 PMC5804390

[11] Breschi L, Mazzoni A, Ruggeri A, Cadenaro M, Di Lenarda R, De Stefano Dorigo E. Dental adhesion review: aging and stability of the bonded interface. Dent Mater. 2008;24:90–101. 17442386 10.1016/j.dental.2007.02.009

[12] Yumitate M, Mine A, Higashi M, Matsumoto M, Hagino R, Ban S, et al. Effect of tooth temperature on the dentin bonding durability of a self-curing adhesives: The discrepancy between the laboratory setting and inside the mouth. Dent Mater J. 2022;41:317–22. 10.4012/dmj.2021-184. 34980768 10.4012/dmj.2021-184

[13] Perdigão J, Araujo E, Ramos RQ, Gomes G, Pizzolotto L. Adhesive dentistry: Current concepts and clinical considerations. J Esthet Restor Dent. 2021;33:51–68. 33264490 10.1111/jerd.12692

[14] Ahmed MH, Yoshihara K, Mercelis B, Van Landuyt K, Peumans M, Van Meerbeek B. Quick bonding using a universal adhesive. Clin Oral Investig. 2020;24:2837–51. 31813057 10.1007/s00784-019-03149-8

[15] Ekambaram M, Yiu CKY, Matinlinna JP. An overview of solvents in resin–dentin bonding. Int J Adhes Adhes. 2015;57:22–33.

[16] Donmez N, Ari H, Belli S. Effect of storage temperature on bond strength of a self-etch adhesive system to pulp chamber dentin. Eur J Dent. 2009;3:314–7. 19826604 PMC2761163

[17] Hardan L, Bourgi R, Kharouf N, Mancino D, Zarow M, Jakubowicz N, et al. Bond strength of universal adhesives to dentin: a systematic review and meta-analysis. Polymers. 2021;13:814. 33799923 10.3390/polym13050814 PMC7961712

[18] Masarwa N, Mohamed A, Abou-Rabii I, Abu Zaghlan R, Steier L. Longevity of self-etch dentin bonding adhesives compared to etch-and-rinse dentin bonding adhesives: a systematic review. J Evid Based Dent Pr. 2016;16:96–106. 10.1016/j.jebdp.2016.03.003 27449836

[19] Mathews IE, Arathi G, Balagopal S. Heat radiation vs air drying to remove interfacial water from self-etch adhesives. Indian J Dent Res. 2008;19:147–9. 18445933 10.4103/0970-9290.40470

[20] Besnault C, Attal JP. Influence of a simulated oral environment on microleakage of two adhesive systems in Class II composite restorations. J Dent. 2002;30:1–6. 11741728 10.1016/s0300-5712(01)00050-1

[21] Li MZ, Wang JR, Liu H, Wang X, Gan K, Liu XJ, et al. Effects of light curing modes and ethanol-wet bonding on dentin bonding properties. J Zhejiang Univ Sci B. 2016;17:703–11. 27604862 10.1631/jzus.B1600055 PMC5018617

[22] de Alexandre RS, Sundfeld RH, Giannini M, Lovadino JR. The influence of temperature of three adhesive systems on bonding to ground enamel. Oper Dent. 2008;33:272–81. 18505217 10.2341/07-79

[23] Reis A, Klein-Júnior CA, Accorinte Mde L, Grande RH, dos Santos CB, Loguercio AD Effects of adhesive temperature on the early and 6-month dentin bonding. J Dent. 2009;37791-798. 10.1016/j.jdent.2009.06.007 19608324

[24] Loguercio AD, Salvalaggio D, Piva AE, Klein-Júnior CA, Accorinte Mde L, Meier MM, et al. Adhesive temperature: effects on adhesive properties and resin-dentin bond strength. Oper Dent. 2011;36:293–303. 21851256 10.2341/10-218L

[25] Borges GA, Spohr AM, de Oliveira WJ, Correr-Sobrinho L, Correr AB, Borges LH. Effect of refrigeration on bond strength of self-etching adhesive systems. Braz Dent J. 2006;17:186–90. 17262122 10.1590/s0103-64402006000300002

[26] Graham JB, Vandewalle KS. Effect of long-term storage temperatures on the bond strength of self-etch adhesives. Mil Med. 2010;175:68–71. 20108846 10.7205/milmed-d-09-00019

[27] Nishiyama N, Tay FR, Fujita K, Pashley DH, Ikemura K, Hiraishi N, et al. Hydrolysis of functional monomers in a single-bottle self-etching primer-correlation of 13C NMR and TEM findings. J Dent Res. 2006;85:422–6. 16632754 10.1177/154405910608500505 PMC2245806

[28] Iliev G, Hardan L, Kassis C, Bourgi R, Cuevas-Suárez CE, Lukomska-Szymanska M, et al. Shelf Life and Storage Conditions of Universal Adhesives: A Literature Review. Polymers. 2021;13:2708. 34451245 10.3390/polym13162708 PMC8400728

[29] Cardoso SA, Oliveira HL, Münchow EA, Carreño NLV, Gonini Junior A, Piva E. Effect of shelf-life simulation on the bond strength of self-etch adhesive systems to dentin. Appl Adhes Sci. 2014;2:26.

[30] Sharafeddin F, Nouri H, Koohpeima F. The effect of temperature on shear bond strength of Clearfil SE bond and adper single bond adhesive systems to dentin. J Dent. 2015;16:10–16. PMC4345108 25759852

[31] Hisamatsu N, Atsuta M, Matsumura H. Effect of silane primers and unfilled resin bonding agents on repair bond strength of a prosthodontic microfilled composite. J Oral Rehabil. 2002;29:644–8. 12153453 10.1046/j.1365-2842.2002.00899.x

[32] Pazinatto FB, Marquezini L Jr, Atta MT. Influence of temperature on the spreading velocity of simplified-step adhesive systems. J Esthet Restor Dent. 2006;18:38–46. 16426509 10.2310/6130.2006.00009

[33] Spohr AM, Correr Sobrinho L, Consani S, Sinhoreti MA, Borges GA. Effect of refrigeration on tensile bond strength of three adhesive systems. Braz Dent J. 2001;12:75–79. 11445917

[34] Reis AF, Oliveira MT, Giannini M, De Goes MF, Rueggeberg FA. The effect of organic solvents on one-bottle adhesives' bond strength to enamel and dentin. Oper Dent. 2003;28:700–6. 14653283

[35] Ikeda T, De Munck J, Shirai K, Hikita K, Inoue S, Sano H, et al. Effect of evaporation of primer components on ultimate tensile strengths of primer-adhesive mixture. Dent Mater. 2005;21:1051–8. 16140370 10.1016/j.dental.2005.03.010

[36] Klein-Júnior CA, Zander-Grande C, Amaral R, Stanislawczuk R, Garcia EJ, Baumhardt-Neto R, et al. Evaporating solvents with a warm air-stream: effects on adhesive layer properties and resin-dentin bond strengths. J Dent. 2008;36:618–25. 18550254 10.1016/j.jdent.2008.04.014

[37] Abate PF, Rodriguez VI, Macchi RL. Evaporation of solvent in one-bottle adhesives. J Dent. 2000;28:437–40. 10856809 10.1016/s0300-5712(00)00018-x

[38] Yiu CK, Pashley EL, Hiraishi N, King NM, Goracci C, Ferrari M, et al. Solvent and water retention in dental adhesive blends after evaporation. Biomaterials. 2005;26:6863–72. 15964621 10.1016/j.biomaterials.2005.05.011

[39] Sundfeld RH, da Silva AM, Croll TP, de Oliveira CH, Briso AL, de Alexandre RS, et al. The effect of temperature on self-etching adhesive penetration. Compend Contin Educ Dent. 2006;27:552–81. 17120389

[40] El-Maksoud OA, Hamama HHH, Wafaie RA, El-Wassefy N, Mahmoud SH. Effect of shelf-storage temperature on degree of conversion and microhardness of composite restorative materials. BMC Oral Health. 2023;23:57. 36721191 10.1186/s12903-023-02770-0 PMC9890862

[41] El-Maksoud OA, Hamama H, Wafaie RA, El-Wassefy N, Mahmoud SH. Impact of refrigeration of different Resin composite restorative materials on the marginal adaptation in class II restorations. BMC Oral Health. 2024;24:1174. 39363215 10.1186/s12903-024-04886-3 PMC11451262

[42] Daronch M, Rueggeberg FA, De Goes MF. Monomer conversion of pre-heated composite. J Dent Res. 2005;84:663–7. 15972598 10.1177/154405910508400716

[43] Hagge MS, Lindemuth JS, Broome JC, Fox MJ. Effect of refrigeration on shear bond strength of three dentin bonding systems. Am J Dent. 1999;12:131–3. 10649935

[44] Asmussen E, Peutzfeldt A. Temperature rise induced by some light emitting diode and quartz-tungsten-halogen curing units. Eur J Oral Sci. 2005;113:96–8. 15693836 10.1111/j.1600-0722.2004.00181.x

[45] Akarsu S, Aktuğ KS. In vitro effect of temperature on dentin bond strength of universal adhesive systems. Odovtos-Int J Dent Sc. 2020;22:93–101.

